# A Case of Laparoscopic Distal Gastrectomy after Failure of Laparoscopic Gastric Antral Devascularization for Gastric Antral Vascular Ectasia

**DOI:** 10.70352/scrj.cr.25-0268

**Published:** 2025-08-22

**Authors:** Kozue Matsuishi, Taisuke Yagi, Ryota Omura, Tomo Horinouchi, Toshihiko Yusa, Takayoshi Kaida, Yuki Kiyozumi, Kenji Shimizu, Kensuke Yamamura, Katsunori Imai

**Affiliations:** Department of Surgery, Saiseikai Kumamoto Hospital, Kumamoto, Japan

**Keywords:** gastric antral vascular ectasia, gastrointestinal hemorrhage, laparoscopic distal gastrectomy

## Abstract

**INTRODUCTION:**

Gastric antral vascular ectasia (GAVE) is a condition characterized by clusters of dilated capillaries in the gastric antrum, leading to gastrointestinal bleeding. Although massive hemorrhage is rare, some cases present with recurrent minor bleeding, which can make endoscopic hemostasis challenging. Here, we report a case of GAVE that was refractory to argon plasma coagulation (APC) and required surgical intervention.

**CASE PRESENTATION:**

A 74-year-old man with end-stage renal disease who was on hemodialysis was referred to our hospital for evaluation of refractory anemia. Upper gastrointestinal endoscopy revealed the characteristic “watermelon stomach” appearance, with radially and longitudinally distributed dilated capillaries in the gastric antrum, leading to a diagnosis of GAVE. Despite undergoing APC 4 times, his anemia persisted. Given the refractory nature of the condition, surgical intervention was considered. To preserve the stomach, we initially performed laparoscopic gastric antral devascularization to reduce the blood flow to the affected area. Intraoperatively, dilated marginal vessels were observed along the greater curvature of the gastric antrum. The marginal artery was ligated along the greater curvature from the watershed area to the pylorus and pyloric ring. Indocyanine green (ICG) fluorescence imaging revealed delayed enhancement in the marginal artery resection area, indicating reduced perfusion. However, after a 2-month postoperative observation period, no improvement in the anemia was observed, and follow-up endoscopy revealed no significant changes in the gastric antral lesions. Consequently, a laparoscopic distal gastrectomy was performed. Following the procedure, the anemia stabilized, and the postoperative course was uneventful.

**CONCLUSIONS:**

Gastric antral devascularization was ineffective for the treatment of GAVE, even when combined with ICG blood flow assessment. For refractory GAVE unresponsive to endoscopic therapy, a distal gastrectomy appears to be the most effective treatment.

## Abbreviations

APC
: argon plasma coagulation
EGD
: esophagogastroduodenoscopy
GAVE
: gastric antral vascular ectasia
ICG
: indocyanine green

## INTRODUCTION

Gastric antral vascular ectasia (GAVE) is a disease characterized by clusters of dilated capillaries in the gastric antrum, leading to gastrointestinal bleeding.^1,2)^ Although massive hemorrhage is rare, intermittent bleeding can occur, sometimes necessitating an endoscopic intervention. Argon plasma coagulation (APC) is the standard endoscopic treatment; however, some cases are refractory to endoscopic hemostasis.^3)^ Here, we report a case of APC-resistant GAVE in which laparoscopic gastric antral devascularization was performed but proved ineffective, and a laparoscopic distal gastrectomy was ultimately required.

## CASE PRESENTATION

**Fig. 1:**
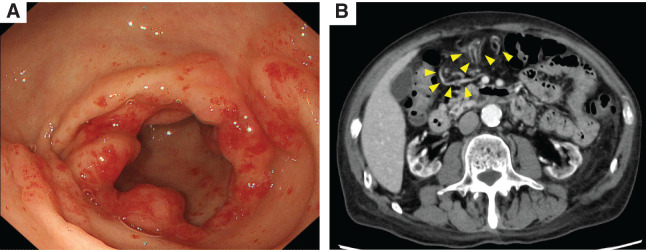
Endoscopic and contrast-enhanced CT findings before surgery. (**A**) EGD showing the characteristic “watermelon stomach” appearance, with prominent erythematous tortuous folds in the antrum, consistent with GAVE. (**B**) Contrast-enhanced CT demonstrating dilated omental arteries and veins. GAVE, gastric antral vascular ectasia; EGD, esophagogastroduodenoscopy

**Fig. 2:**
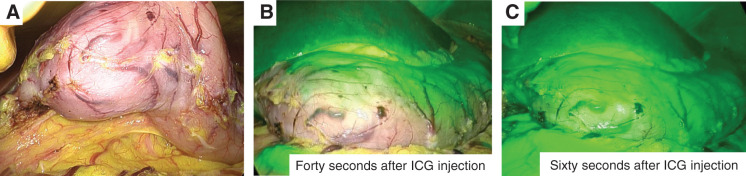
Intraoperative findings for gastric antral devascularization. (**A**) The omental artery and vein were dissected along the stomach wall, detaching the omental attachment. (**B**) Blood flow assessment using ICG fluorescence imaging at 40 s after ICG injection. (**C**) ICG fluorescence imaging at 60 s after ICG injection. ICG, indocyanine green

**Fig. 3:**
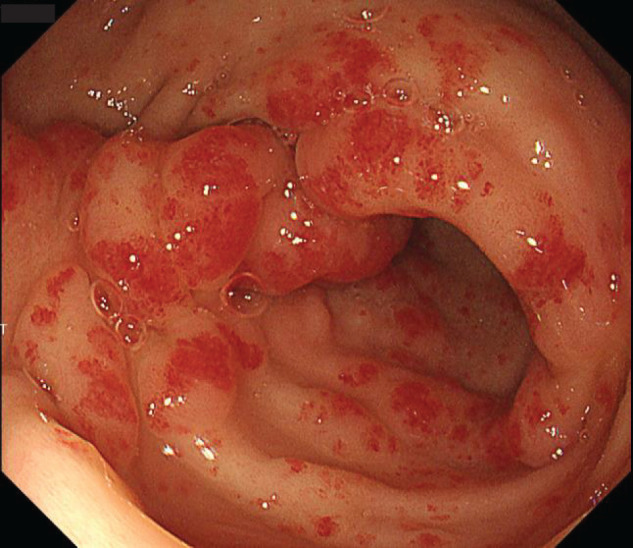
Endoscopic findings of the GAVE lesions. The endoscopic findings after the vascular resection are shown. GAVE, gastric antral vascular ectasia

**Fig. 4:**
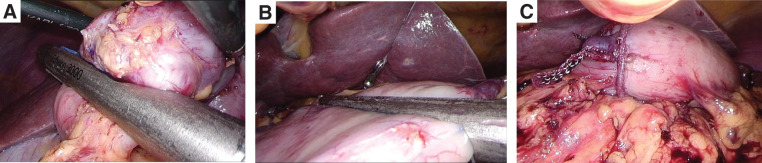
Distal gastrectomy. (**A**) Resection of the duodenum using a 60-mm blue cartridge linear stapler. (**B**) Resection of the stomach using two 60-mm blue cartridge linear staplers. (**C**) Reconstruction using a delta-shaped anastomosis with Billroth I reconstruction using 45- and 60-mm blue cartridge staplers.

A 74-year-old man with end-stage renal disease secondary to glomerulonephritis was undergoing maintenance hemodialysis. He had a history of chronic renal anemia. However, his hemoglobin level decreased to 5.3 g/dL, and a fecal occult blood test performed at a previous hospital was positive. As gastrointestinal bleeding was suspected, he was referred to our institution for further evaluation. Esophagogastroduodenoscopy (EGD) revealed the characteristic “watermelon stomach” appearance with radially and longitudinally distributed dilated capillaries in the gastric antrum, leading to a diagnosis of GAVE (**Fig. 1A**). No active bleeding was observed during the EGD, and lower gastrointestinal endoscopy and small-bowel capsule endoscopy failed to identify an alternative bleeding source. Contrast-enhanced CT did not reveal any definitive source of bleeding but demonstrated mild dilation of the right gastroepiploic artery and vein (**Fig. 1B**). Based on these findings, his anemia was suspected to be caused by bleeding from the GAVE lesions, and treatment with APC was performed. Although APC was conducted 4 times, the vasodilation persisted, and the patient required repeated blood transfusions for anemia. Consequently, a surgical intervention was recommended. Although a gastrectomy was considered, the patient strongly preferred to preserve the stomach. Therefore, we decided to perform vascular decompression from the viewpoint of organ preservation, and laparoscopic gastric antral devascularization was carried out. An intraperitoneal inspection revealed dilation of the marginal vessels along the greater curvature of the gastric antrum. The marginal artery and vein were ligated and divided along the greater curvature from the watershed area to the pylorus and pyloric ring. The blood flow in the lesser curvature was preserved because of concerns about ischemia in the pylorus (**Fig. 2A**). Indocyanine green (ICG) fluorescence imaging revealed delayed enhancement in the marginal artery resection area, indicating reduced perfusion (**Fig. 2B** and **2C**). There was concern that the complete absence of enhancement could lead to complications such as partial gastric wall necrosis. Therefore, the procedure was concluded with the expectation of reduced perfusion, and the postoperative course was managed with close monitoring of anemia progression. The postoperative course was uneventful, and the patient was discharged on POD 5. However, during outpatient follow-up, his anemia persisted, necessitating frequent blood transfusions. Follow-up EGD showed no improvement in the GAVE lesions (**Fig. 3**). Therefore, a laparoscopic distal gastrectomy was ultimately performed at 3 months after the initial surgery. Intraoperatively, the right gastroepiploic artery and vein had already been divided from the gastric angle along the greater curvature to the duodenal bulb during the initial procedure. The right gastric artery and vein, along with the superior duodenal artery, were subsequently dissected along the gastric wall. The duodenum was transected using a 60-mm blue cartridge linear stapler (ECHELON 3000 Stapler; Johnson & Johnson, New Brunswick, NJ, USA) (**Fig. 4A**), and the stomach was resected near the gastric angle using two 60-mm blue cartridge linear staplers (**Fig. 4B**), with removal of the resected specimen. A Billroth I reconstruction was performed with a delta-shaped anastomosis using 45- and 60-mm blue cartridge staplers (**Fig. 4C**). The total operative time was 196 min, with an estimated blood loss of 200 g.

**Fig. 5:**
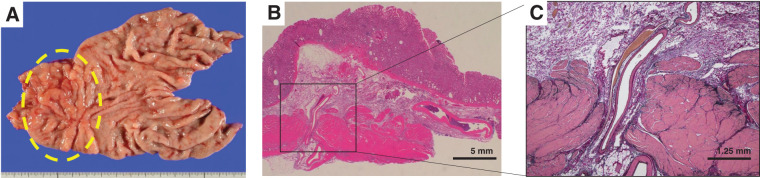
Surgical specimen and histological findings (hematoxylin–eosin and immunohistochemical staining). (**A**) Photograph of the resected specimen showing prominent reddish telangiectasia in the antrum. (**B**) Histopathological examination revealing dilated capillaries and arteries within the submucosa, along with arteries and veins penetrating the muscularis propria from the subserosal layer (original magnification, ×5). (**C**) Elastica van Gieson staining highlighting the dilated arteries and veins within the submucosa, indicative of a complex arteriovenous malformation (original magnification, ×20).

Macroscopic examination of the resected specimen revealed a reddish lesion in the gastric antrum (**Fig. 5A**). Pathological findings demonstrated sporadic capillary proliferation within the lamina propria mucosa and telangiectasia, as well as multiple small and large capillaries in the submucosa (**Fig. 5B**). In certain areas, dilated arteries and veins extended from the subserosa through the muscularis propria into the submucosa, suggestive of an arteriovenous malformation. The final pathological diagnosis was a complex vascular malformation (**Fig. 5C**). The postoperative course was uneventful, and the patient was discharged on POD 6. No recurrence of anemia has been observed since the procedure.

## DISCUSSION

GAVE accounts for approximately 4% of non-variceal upper gastrointestinal bleeding cases and is characterized endoscopically by a distinctive “watermelon stomach” appearance due to radially distributed dilated capillaries in the gastric antrum.^1,2)^ The pathogenesis of GAVE remains incompletely understood, but several mechanisms have been proposed, including antral hyperperistalsis leading to pyloric prolapse, vasodilatory effects of gastrin, autoimmune factors, portal hypertension, and metabolic derangements associated with renal failure.^4)^ While GAVE can lead to chronic gastrointestinal bleeding and subsequent anemia, active bleeding is infrequently observed during endoscopic examination. Although GAVE is most commonly associated with liver cirrhosis,^5)^ a wide range of other comorbidities, including autoimmune disorders,^6)^ cardiac diseases,^7)^ and chronic kidney disease,^8)^ have also been reported.

Minimally invasive endoscopic therapies have become the first-line treatment for GAVE. Various thermal coagulation techniques have shown efficacy for GAVE, including laser photocoagulation,^9)^ radiofrequency ablation,^10)^ the heat probe method,^11)^ and APC.^12–14)^ Among these, APC is widely favored due to its ease of use, ability to coagulate large areas, and high therapeutic efficacy. However, recurrence and rebleeding are common, necessitating repeated interventions in many cases.^15,16)^

For cases refractory to endoscopic therapy, a surgical intervention may be required.^17,18)^ We performed a literature review using the terms “gastric antral vascular ectasia,” “GAVE,” “surgery,” and “gastrectomy” in the PubMed database and identified 12 surgical cases of APC-resistant GAVE (**Table 1**).^2,12,17–26)^ The surgical treatment for GAVE was gastrectomy in all cases. Four patients had liver disease, and 3 had renal disease. Bleeding was successfully controlled in all patients after surgery. In the present case, the patient exhibited resistance to endoscopic treatment, necessitating a surgical intervention. In this case, contrast CT showed dilation of the right gastroepiploic artery and vein, and we hypothesized that the cause may have been the development of blood vessels in the antrum. Inspired by the Hassab procedure for gastroesophageal varices,^27)^ we devised a technique for vascular devascularization of the gastric antrum. However, because there are no previous reports of laparoscopic devascularization for GAVE without concomitant gastrectomy in the PubMed database, the efficacy of antral vascular devascularization could not be confirmed. Given the patient’s strong preference for preservation of the stomach, we chose to perform a less invasive occlusion rather than a gastrectomy. However, the anemia persisted postoperatively, and follow-up EGD showed no improvement in the GAVE lesions. The limited efficacy of devascularization in this case may be attributable to the rich intramural vascular network of the stomach,^28)^ resulting in insufficient reduction of the blood flow at the resection site. Histopathological examination of the resected specimen revealed capillary proliferation and the presence of arteriovenous anastomoses within the lesions. These findings suggest that ligation of the right gastroepiploic artery and vein alone may not have adequately disrupted the antral capillary blood supply. Because resection of the blood vessels on the lesser curvature side, which is the reflux area of the right gastric artery, could have caused ischemia in the pylorus, we considered that treatment by vascular blockade would be difficult, and therefore chose a distal gastrectomy.

**Table 1:** Twelve surgical cases of APC-resistant GAVE

Case	Author	Age	Sex	Comorbidity	Endoscopic findings	Initial treatment	Surgical procedure
1	Jouanolle et al.^19)^	66	F	Systemic sclerosis	Watermelon	APC	ODG
2	Probst et al.^12)^	51	F	None	Watermelon	APC	ODG
3	Sherman et al.^18)^	78	F	None	Watermelon	APC	LADG
4	Pljesa et al.^20)^	54	F	Chronic pyelonephritis	Watermelon	APC and EBL	OTG
5	Belle et al.^21)^	71	F	None	—	APC	LADG
6	Yildiz et al.^22)^	62	F	None	Watermelon	APC	ODG
7	Jin et al.^2)^	75	M	None	Watermelon	APC	ODG
8	Becq et al.^23)^	67	M	Cirrhosis	—	APC and TIPS	ODG
9	Lim et al.^24)^	56	F	DVT, PTE	Watermelon	APC	LADG
10	Alsaeed et al.^17)^	54	F	ESRD	Watermelon	APC	LAsTG
11	Itagaki et al.^25)^	62	M	Cirrhosis, diabetes, ESRD	DAVE	APC	ODG
12	Girão de Caires et al.^26)^	75	F	Chronic hepatitis	Watermelon	APC and EBL	ODG

APC, argon plasma coagulation; DAVE, diffuse gastric antral vascular ectasia; DVT, deep vein thrombosis; EBL, endoscopic band ligation; ESRD, end-stage renal disease; F, female; GAVE, gastric antral vascular ectasia; LADG, laparoscopy-assisted distal gastrectomy; LAsTG, laparoscopic assisted subtotal gastrectomy; M, male; ODG, open distal gastrectomy; OTG, open total gastrectomy; PTE, pulmonary thromboembolism; TIPS, transjugular intrahepatic portosystemic shunt

Pathologically, GAVE is characterized by capillary dilation and proliferation in the lamina propria, thrombotic occlusion within capillaries, and fibromuscular hyperplasia.^29)^ The lesions can extend beyond the lamina propria into the submucosa, and this may contribute to the high recurrence rate after endoscopic treatment of the mucosal surface alone. In the present case, a histological examination revealed dilated arteries and veins extending from the subserosa through the muscularis propria into the submucosa, suggestive of an underlying arteriovenous malformation. Although dilated capillaries deep in the submucosa have been described in APC-refractory cases,^30)^ there are no reports of abnormal blood vessels extending from the subserosa through the muscularis propria to the submucosa. The present case demonstrated the characteristic features of GAVE, including dilated capillaries within the lamina propria and submucosa. In addition to these findings, paired arteries and veins penetrating from the serosa through the muscularis propria were observed in deeper layers. These vessel structures exhibited arteriovenous transition, consistent with a vascular malformation. Accordingly, from a pathological perspective, this lesion can be classified as a form of vascular malformation in a broad sense. While the exact etiology of GAVE remains unclear, the presence of such vascular anomalies may be associated with cases that are refractory to endoscopic therapies such as APC.

## CONCLUSIONS

For GAVE cases that are refractory to endoscopic treatment, devascularization alone may not provide sufficient therapeutic benefits. The present case highlights the potential limitations of devascularization and supports the efficacy of a distal gastrectomy as the definitive treatment option, leading to a favorable clinical outcome.

## DECLARATIONS

### Availability of data and materials

All data generated or analyzed during this study are included in the published article.
